# Detoxification Cytochrome P450s (CYPs) in Families 1–3 Produce Functional Oxylipins from Polyunsaturated Fatty Acids

**DOI:** 10.3390/cells12010082

**Published:** 2022-12-24

**Authors:** Jazmine A. Eccles, William S. Baldwin

**Affiliations:** Department of Biological Sciences, Clemson University, Clemson, SC 29634, USA

**Keywords:** oxylipin, cytochrome P450 (CYP), inflammation, adverse drug reaction, inhibition, obesity, lipid metabolism, PUFA, hormone, PPAR

## Abstract

This manuscript reviews the CYP-mediated production of oxylipins and the current known function of these diverse set of oxylipins with emphasis on the detoxification CYPs in families 1–3. Our knowledge of oxylipin function has greatly increased over the past 3–7 years with new theories on stability and function. This includes a significant amount of new information on oxylipins produced from linoleic acid (LA) and the omega-3 PUFA-derived oxylipins such as α-linolenic acid (ALA), docosahexaenoic acid (DHA), and eicosapentaenoic acid (EPA). However, there is still a lack of knowledge regarding the primary CYP responsible for producing specific oxylipins, and a lack of mechanistic insight for some clinical associations between outcomes and oxylipin levels. In addition, the role of CYPs in the production of oxylipins as signaling molecules for obesity, energy utilization, and development have increased greatly with potential interactions between diet, endocrinology, and pharmacology/toxicology due to nuclear receptor mediated CYP induction, CYP inhibition, and receptor interactions/crosstalk. The potential for diet-diet and diet-drug/chemical interactions is high given that these promiscuous CYPs metabolize a plethora of different endogenous and exogenous chemicals.

## 1. Introduction

### 1.1. Background

Dietary lipids provide energy utilization, structure, and signaling. These lipids can be divided into saturated fatty acids (SAFAs), polyunsaturated fatty acids (PUFAs) and monounsaturated fatty acids (MUFAs; n-9). The PUFAs can be further divided into n-3 (omega-3) and n-6 (omega-6) fatty acids of which oxylipins are derived. Many of the PUFAs and their oxylipin derivatives have multiple diverse purposes, including inflammation, pain, cell adhesion, energy distribution and use, angiogenesis, apoptosis, blood pressure, hunger, blood coagulation, and more [1,2,3,4,5]. The PUFAs and their oxylipin derivatives can directly interact with a number of receptors in multiple tissues and enhance lipid signaling. These functions may be highly specific or relate directly to nutrition by aiding the distribution and use of fats [2,6,7].

**Figure 1:**
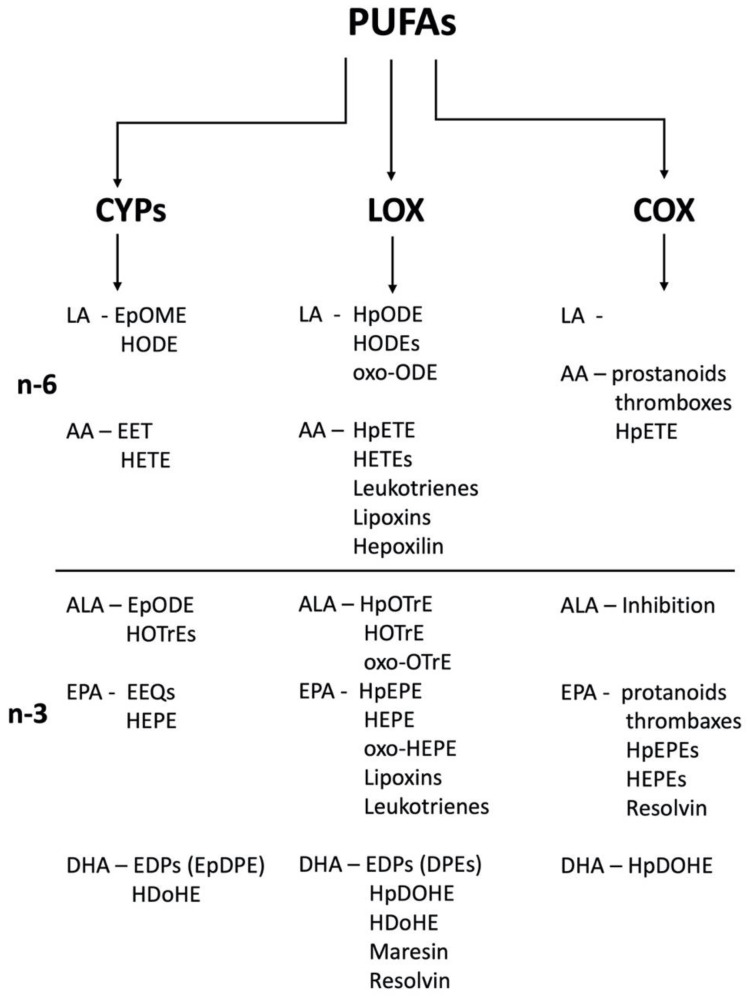
PUFAs are primarily metabolized the CYPs, lipoxygenases (LOX), and cyclooxygenases (COX) with overlapping oxylipin biosynthesis capabilities. LA = linoleic acid (18:2); AA = arachidonic acid (20:4); ALA = α-linolenic acid (18:3); EPA = eicosapentaenoic acid (20:5); DHA = docosahexaenoic acid (22:6).

PUFAs can be metabolized to multiple oxylipins by the cyclooxygenase (COX), lipoxygenase (LOX), and or cytochrome P450 (CYP) pathways (Figure 1). Products produced include the thromboxanes, prostaglandins, leukotrienes, lipoxins, and the less studied and more recently measured CYP-derived oxylipins such as the epoxides and diols produced from those epoxides by soluble epoxide hydrolase (sEH). These oxylipins may have powerful activity at multiple receptors [3]; however, some oxylipins have little function or no verified function and the roles of several oxylipins have been poorly defined [1,8].

Oxylipins can be derived from n-3 and n-6 PUFAs (Figure 1). The n-3 PUFAs are often considered anti-inflammatory and the n-6 PUFAs inflammatory, but that is an overly simplistic characterization roughly based on the mostly inflammatory properties of arachidonic acid (AA) and linoleic acid (LA), their metabolites, and the activity of the non-steroidal anti-inflammatory drugs (NSAIDs) that primarily inhibit cyclooxygenases and block the production of prostaglandins produced from arachidonic acid. This is coupled with the specific activities of several n-3 PUFAs such as docosahexaenoic acid (DHA) and eicosapentaenoic acid (EPA) involved in development, vascularization and other biological activity. In addition, Fat-1 transgenic mice that can convert n-6 PUFAs to n-3 PUFAs show improved cardiovascular health, insulin sensitivity, and reproduction [9,10]; however, other studies demonstrate that n-6 fatty acids are also crucial in reproductive health [11,12]. There is concern that modern diets have increased the ingested ratio of n-6:n-3 PUFAs and in turn led to increased inflammation, cancer, obesity, cardiovascular disease and other modern afflictions of over-consumption enhanced by the lack of fresh fruits and vegetables [13,14].

PUFAs, as parent compounds or following oxidative metabolism, can bind to and activate multiple receptors, including the peroxisome proliferator-activated receptors (PPARs), hepatocyte nuclear factors (HNF4α), and a large number of G-protein coupled membrane bound receptors (GPCRs) [6,8,15,16,17]. In turn, PUFAs mediate adipocyte differentiation, lipid uptake, metabolism, or storage depending on the PPAR activated or tissue involved; non-insulin dependent diabetes and inflammation through HNF4α, and inflammation, diabetes, obesity, pain regulation, and hypertension through multiple other receptors, including GPCRs [6,15,18].

This review will focus on the oxylipins derived from detoxification CYPs in families 1–3. New analytical techniques have allowed for increased sensitivity and therefore the measurement of greater numbers of oxylipins, including the CYP-derived oxylipins. Furthermore, analytical and molecular techniques have allowed for greater mechanistic understanding of their effects in multiple cells and tissues. Last, these CYPs are highly inducible, some are gender predominant, and most have multiple functions in the body such as bile acid, xenobiotic, steroid, and fatty acid metabolism. This can lead to differential metabolism depending on diet, sex, environmental influences, pharmaceutical use, and potentially unexpected consequences. Taken together, further study of the detoxification CYP-mediated oxylipins is needed in order to understand their function and how these functions might be perturbed.

### 1.2. CYPs and Changes in CYP Expression and Activity

CYPs are phase I enzymes that mono-oxygenate, reduce, and hydrolyze substrates thus making active molecules that are often more polar and easier to conjugate by phase II enzymes for rapid removal from the body [19]. They are often key detoxification enzymes in the liver and provide protection from xenobiotics and endobiotics. The CYPs are grouped into families, subfamilies and isoforms. For example, there are 57 human CYPs arranged into 18 families and 43 subfamilies [20]. Each CYP is named based on its family number first, followed by a letter to indicate the subfamily, and then a number that indicates the gene. For example CYP3A4 is a human CYP in the “third” family, “A” subfamily, gene “4”. It is the CYPs in families 1–3 that contribute the most to the metabolism of environmental contaminants and pharmaceuticals [21,22,23].

In general, because the purified CYPs are from human genes our specific knowledge of oxylipins produced from individual CYPs is best understood in humans. Epidemiological data provides some basis for our understanding of the function of the CYP-derived oxylipins, but often mouse and sometimes rat models inform our understanding of oxylipin function. Several humanized mouse models have also helped provide key data on the function of human CYPs in the production and function of oxylipins. When possible this review focuses mostly on human data but not exclusively. Human data is often presented in the tables sometimes with evidence from mice in the corresponding paragraphs. CYP nomenclature is based on homology and therefore most, but not all CYPs have unique names and thus different names from their homologous families in other mammalian species [20]. There may be rare cases where it is not clear which species are being discussed and therefore we provided a table of the common individual isoforms found in each species (human, mouse, rat) by family (Table 1) [20].

Liver CYP expression is highly variable based on both genetic polymorphisms and inducibility by diet and chemical exposure [24,25,26,27,28,29]. Human CYP2D6 and to a lesser extent CYP2B6 are highly polymorphic with variants that perturb drug (and anandamide) metabolism [25,30,31,32,33,34]. Several transcription factors are xenosensors that induce CYP expression in order to acclimate to current levels of xeno- and endobiotic chemicals, including the Aryl hydrocarbon receptor (AhR), pregnane X-receptor (PXR), constitutive androstane receptor (CAR), and peroxisome proliferator-activated receptors (PPARs) [19,27,35]. Thus, drug metabolites, hormones, and oxylipin levels can all be mediated by differences in expression and activity of these enzymes. This also means that inhibition of CYPs by drugs, pesticides, dietary sources or other endobiotics can affect metabolism [36,37,38].

Several CYPs are regulated by growth hormone release patterns in a male or female specific or predominant manner with help from HNF4α including Cyp2b9, Cyp2b10, Cyp2b13, Cyp3a41, Cyp3a44, Cyp2d9 and others [39,40,41,42]. Xenobiotic and diet-mediated sexually dimorphic induction has also been observed for Cyp2a4, Cyp2c40, Cyp2b9 and other murine CYPs [41,42,43,44], suggesting that the sexually dimorphic expression of CYPs and subsequent metabolism of endobiotics may play a role in sexually dimorphic disease.

For example, several of the obesogenic or anti-obesogenic effects of CYPs are gender predominant (see below) [44,45,46,47]. Androgen-dependent induction of CYP4A8 and CYP4A12, preferentially in males, leads to increased 20-HETE production from arachidonic acid and increased hypertension [48,49]. The female predominant Cyp2c29 in mice produces 12,13- and 14,15-EET and in turn increases vasodilation, potentially in an estrogen-dependent manner [50]. Furthermore, increased blood pressure caused by the loss of Cyp2j5 function in -nullizygous mice is female specific and indicates the importance of this enzyme in the production of EETs in female kidneys [51]. Last, cardiac ischemic injury increases during peri-menopause in association with significant changes in the oxylipin profile, especially the 9,10- and 12,13-EpOME/DiHOME ratios [52]. Taken together, changes in CYP expression including sexually dimorphic CYP expression can effect oxylipin production and disease progression.

### 1.3. CYP Expression, Obesity, and Oxylipins

Interestingly, several xenobiotic-metabolizing CYPs are associated with obesity and related metabolic diseases in mice. For example, Cyp2c-null mice that lack 14 of the 15 Cyp2c isoforms are resistant to high-fat diet induced obesity in males [46]. The loss of Cyp3a expression, the most highly expressed subfamily of liver CYPs, reduced high-fat diet induced obesity in female mice only [47]. Cyp3a inhibitors such as grapefruit juice (naringin) are also associated with reduced adiposity and weight gain in humans and mice coupled with increased *Cpt1a* expression, increased *Ppara* activation and reduced Srebp-1 activity [53,54].

In contrast, Cyp2a5-null mice are more sensitive to diet-induced obesity than WT mice with *Ppara* activity associated with greater obesity but lower steatosis [55]. Furthermore, three human CYP2A6 single nucleotide polymorphisms are associated with obesity providing further evidence that the lack of CYP2A6 is obesogenic [55].

Human CYP2B6 is also inversely associated with obesity as humans with low expression are more likely to be obese [56]. Further evidence is provided by Cyp2b-null mice. Mice that lack the three primarily hepatic Cyp2b members, Cyp2b9, Cyp2b10, and Cyp2b13 (Cyp2b-null or Cyp2b9/10/13-null) show greater susceptibility to high-fat diet induced obesity coupled with increased steatosis in males [44,57]. The presence of human CYP2B6 in Cyp2b-null mice (hCYP2B6-Tg) reduced obesity in the females; however suprisingly, human CYP2B6 increased steatosis in association with several oxylipins including 9-HODE and 13-KODE from linoleic acid, and 12,13-DHET, 14,15-EET, and 14,15-DHET from arachidonic acid [8]. Whether these oxylipins are directly involved in obesity or steatosis is unknown. In agreement, changes in linoleic acid metabolism in hepatic P450 oxidoreductase-null mice are also associated with steatosis and Cyp2b10 induction [58]. Interestingly, a number of LA and ALA oxylipins are associated with obesity and CYP induction following a high soybean oil high-fat diet. These include hepatic but not plasma 9,10-,12,13-, and 15,16-oxylipins from ALA and LA [2].

Overall, these data provide examples of changes in CYP expression and metabolism of PUFAs and subsequent production of oxylipins that are consistent with perturbations in energy metabolism, lipid metabolism, lipid distribution, metabolic disease, and obesity.

## 2. Oxylipin Production by CYPs

Oxylipins are derived from PUFAs by the cyclooxygenase (COX), lipoxygenase (LOX), and or cytochrome P450 (CYP) pathways. Of these, the CYP pathways are dependent and can be easily altered by diet [44,55,59,60,61]. In general, CYP2B, CYP2C, and CYP2J subfamily members are involved in making epoxides; CYP1A, CYP4A, and CYP4F subfamilies are involved in making omega-hydroxylated products from PUFAs [62]. Synthesis of mid-chain HETEs or HODEs is primarily LOX-mediated but may be metabolized by CYP1B1, CYP4A isoforms, or CYP2B members [62,63]. We will take a look at the production of several oxylipins by the CYPs in families 1–3, their activity, and mechanism when known. Table 2 outlines the basic types of oxylipins produced from each type of PUFA covered in this review to provide some basic background before investigating the metabolism of several PUFAs by CYPs.

### 2.1. Linoleic Acid Metabolism

- CYPs primarily metabolize LA into the epoxinated EpOMEs that will be further metabolized by sEH into the DiHOMEs. HpODEs and HODEs may also be produced.
- LA oxylipins activate nuclear and cytosolic receptors such as PPARγ, GPR132, G2A, and TRPV1.
- In turn, most LA-oxylipins are pro-inflammatory, but anti-inflammatory effects potentially mediated by PPARγ have also been observed.

Linoleic acid (LA; 18:2) is an n-6 PUFA comprised of an 18-carbon chain with two double bonds. It is the most highly consumed PUFA in the human diet and is considered an essential fatty acid, meaning humans cannot synthesize it and must consume it [64]. There are a wide variety of sources of LA, but some of the most common foods with high concentrations in the human diet include vegetable oils, seeds, eggs, nuts, and many meats [64,65].

**Figure 2:**
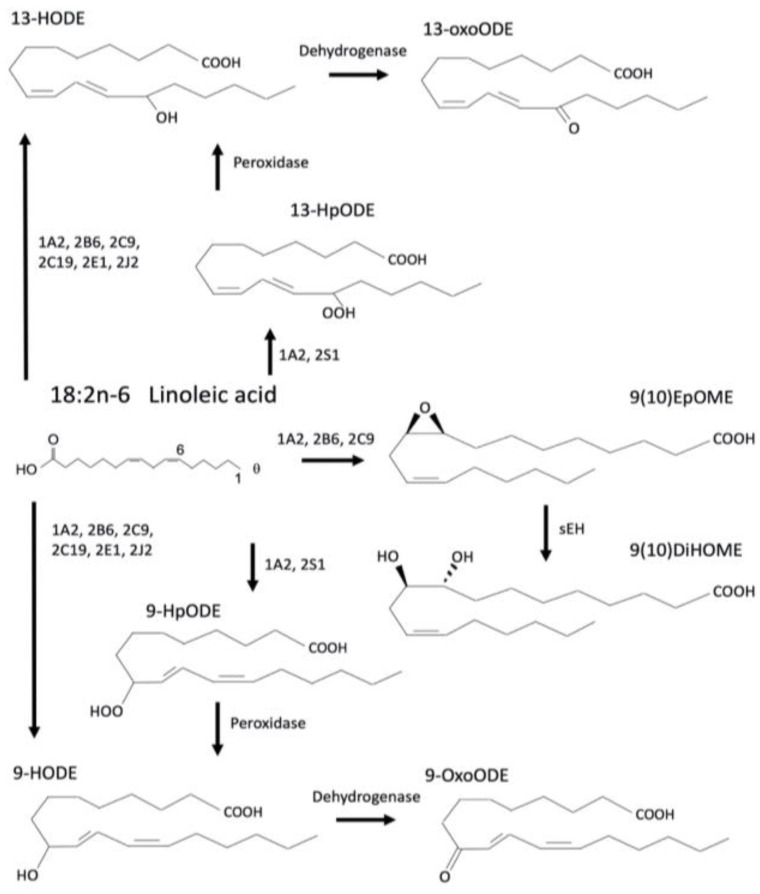
Metabolism of linoleic acid by CYPs produces multiple oxylipins. These oxylipins may be subsequently metabolized by soluble epoxide hydrolase (sEH) or dehydrogenases. Oxylipins include 9-HODE, 13-HODE, 9-HpODE, 13-HpODE, 12,13-EpOME and others that are not shown.

As an essential PUFA, LA can be converted to AA and other n-6 PUFAs [64] or can be metabolized to a variety of oxylipin metabolites including oxidized LA metabolites (OXLAMs) [62] and epoxyoctadecamonoenoic acids (EpOMES) [66]. These can be further metabolized by other reactions including by enzymes such as soluble epoxide hydrolases (sEH), peroxidases and dehydrogenases [66] (Figure 2). The OXLAMs also include the metabolites 9- and 13-hydroxy-octadecadienoic acid (HODE) that can be further metabolized by a dehydrogenase to 9- and 13-oxo-octadecadienoic acid (oxoODE or KODE) [67].

The first step in LA metabolism to the OXLAMs by CYPs is their metabolism to hydroperoxy-octadecadienoic acids (HpODEs) by enzymes such as CYP1A2 [68] and CYP2S1 [69]. This metabolism can occur at the 9 or 13 positions, resulting in the formation of 9- or 13-HpODE. 9-HpODE has been demonstrated to increase glutathione (GSH) oxidation [70], indicating a possible role in oxidative stress. 13-HpODE also induces cellular stress such as increasing smooth muscle cytotoxicity by activating NAD(P)H oxidase [71], or inducing tumor necrosis factor alpha (TNFα), monocyte chemoattractant protein-1 (MCP-1), and granzyme B (GZMB) in Natural Killer (NK) cells [72].

Following the formation of the HpODEs, these oxylipins can be further metabolized by peroxidases to the HODEs. The HODEs can also be directly synthesized from LA, skipping the formation of HpODEs by a variety of CYPs, including 1A2, 2B6, 2C9, 2C19, 2E1, 2J2, and 3A4 [73,74,75,76]. For example, Cyp3a subfamily members produce a number of epoxidated products of linoleic acid and arachidonic acid in human and rodents. CYP3A4 primarily metabolizes linoleic acid to 11-HODE, and the production of 11-HODE is increased 10X by dexamethasone (PXR activator and CYP3A inducer) treatment in rats [75]. OXLAMS are also found in the brain and the production or delivery of OXLAMS without vitamin E causes encephalomalacia and ataxia [3]. Increased 13-HODE reduced platelet aggregation, and beneficially, is involved in early life neuronal morphogenesis during day 0–day 1 in rat cortical neurons [77]. See Table 3 for a summary of the actions of LA-derived oxylipins.

9-HODE has been shown to act as a ligand for PPARγ2 and stimulate fat accumulation [76]. 9-HODE is also a ligand for other receptors, including GPR132 which is involved in sensing and responding to oxidative stress [67] and G2A, a oxidative stress-reactive GPCR found in the skin [78].

13-HODE has been shown to stimulate prostacyclin production by increasing arachidonic acid release [79]. 13-HODE can also act as a ligand for PPARγ [80] and regulate gene expression. Both 9- and 13-HODE regulate fatty acid binding protein 4 (FABP4) expression in macrophages [67], and both are also found at increased concentrations after ischemic stroke, possibly promoting increased inflammation for healing [81] although PPARγ activation is often considered anti-inflammatory. For example, 13-HODE inhibits Leukotriene B4 (LtB4) secretion from stimulated leukocytes, resulting in a reduced inflammatory response [82]. Humanized CYP2B6-Tg mice produce 9-HODE and 13-HODE at greater levels than Cyp2b-null mice. This is associated with reduced diet-induced obesity, but also increased steatosis [8]. The level of HODEs has also been shown to decrease in response to ischemia in wildtype mice and mice that overexpress endothelial CYP2J2 [83], but the implications of this is not known. Further research is needed to understand an exact role and mechanism of action for the HODEs. In addition, as the HODEs are also produced by LOXs, the mechanism for production under differing conditions is often unknown.

**Table 3:** Metabolism of linoleic acid produces several oxylipins with a diverse set of putative functions.

Oxylipin	CAS Number	CYPs/Enzymes	References	Effects	References
9,10-EpOME	6814-52-4	1A2, 2B6, 2C9	[8,84]	Activate NF-kB and AP-1 in endothelial cells resulting in oxidative stress Inhibit osteoblast differentiation through PPARγ2 Obesity	[2,76,85]
9,10-DiHOME	263399-34-4	sEH		Promotes adipogenesis and inhibits osteogenesis through PPARγ2	[76]
12,13-EpOME	Not found	1A2, 2C9, 2E1, 2J2,	[68,84]	Activate NF-kB and AP-1 in endothelial cells resulting in oxidative stress Obesity	[2,85]
12,13-DiHOME	263399-35-5	sEH		Stimulates brown adipose tissue activity in response to cold exposure Stimulates cell proliferation in MCF-7 breast cancer cells Cause mitochondrial dysfunction through activation of the permeability transition Increases exercise-mediated fatty acid uptake Increases sensitivity to thermal pain Cardiac/ischemic injury	[7,16,52,86,87,88]
9-HpODE	63121-49-3	1A2, 2S1	[68,69]	Increases GSH oxidation	[70]
9-HODE	98524-19-7	1A2, 2B6, 2C9, 2C19, 2E1, 2J2 Peroxidase	[8,73,75]	Stimulates fat accumulation through PPARγ2 Associated with reduced obesity, greater glucose sensitivity, but also liver steatosis Decreased in response to ischemia	[8,76,83]
9-oxoODE	54232-59-6	Dehydrogenase		May contribute to pain and hyperalgesia through TRVP1	[89]
13-HpODE	23017-93-8	1A2, 2S1	[68,69]	Can induce smooth muscle cytotoxicity by activating NAD(P)H oxidase Induce TNFα, MCP1, and GZMB in Natural Killer (NK) cells	[71,72]
13-HODE	18104-45-5	1A2, 2B6, 2C9, 2C19, 2E1, 2J2 Peroxidase	[8,75,90]	Stimulates prostacyclin production by increasing arachidonic acid release Decreased in response to ischemia Can inhibit platelet adhesion to endothelial cells Ligand for PPARγ Inhibit LtB4 secretion from stimulated leukocytes	[79,80,82,83,91]
13-oxoODE	29623-29-8	Dehydrogenase		Regulates gene expression in macrophages through PPARγ Reduces IL-8 secretion and has anti-inflammatory effects in colonic epithelial cells Associated with reduced obesity in females; greater glucose sensitivity and liver steatosis in male hCYP2B6-Tg mice	[8,92,93]

The HODEs can then be further metabolized by dehydrogenases to the oxoODEs, but unlike the HODEs, oxoODEs cannot be directly synthesized by CYPs. 9-oxoODE may act on transient receptor potential vanilloid type 1 ion channel (TRPV1) to contribute to pain and hyperalgesia [89]. 13-oxoODE, like 13-HODE, is able to activate PPARγ and regulate gene expression in macrophages [92]. It also reduces IL-8 secretion through PPARγ sginaling and has anti-inflammatory effects in colonic epithelial cells [93]. However, the oxoODEs also have negative consequences. 9- and 13-oxoODE have been implicated in a variety of pathological diseases including non-alcoholic steatohepatitis (NASH) [94] and coronary artery disease [95].

In addition to the OXLAMs, LA can be metabolized into oxylipins called EpOMEs by CYPs. These compounds are the more canonical pathway for production of oxylipins by CYPs. The EpOMEs include 9,10- and 12,13-EpOME. 9,10-EpOME can act on several receptors including PPARγ2 to inhibit osteoblast differentiation [76] and NF-κB and AP-1 to induce oxidative stress in endothelial cells [85]. 12,13-EpOME can also act on NF-κB and AP-1 in the same way 9,10-EpOME does to induce oxidative stress.

The EpOMEs can be further metabolized by sEH to the dihydroxyoctadecenoic acids (DiHOMEs), which include 9,10- and 12,13-DiHOME. 9,10-DiHOME can promote adipogenesis and inhibit osteogenesis through PPARγ2 [76], similarly to 9,10-EpOME. 12,13-DiHOME has several known actions, including stimulating brown adipose tissue activity in response to cold exposure [86], increasing fatty acid uptake in response to exercise [7], increasing sensitization to thermal pain through TRPV1 [16], cardiac ischemic injury [52], stimulating cell proliferation in MCF-7 breast cancer cells [87], and causing mitochondrial dysfunction through activating permeability transition [88]. In summary, the EpOMEs, DiHOMEs, HODEs, and oxoODES produced from LA activate several different receptors, including both nuclear and membrane bound receptors such as PPARγ and TRPV1 as well as other GPCRs, and initiate multiple functions depending on the tissue.

### 2.2. Arachidonic Acid Metabolism

- AA is metabolized by the CYPs to a number of distinct oxylipins including the HETEs and the EETs that are subsequently metabolized by sEH into the DiHETs (also seen as DHETs).
- There are a large number of AA oxylipins that activate a number of GPCRs or act as second messengers
- AA-oxylipins are involved in a variety of processes, including inflammation, vascularization, vasoconstriction, oxidative stress, and apoptosis

**Figure 3:**
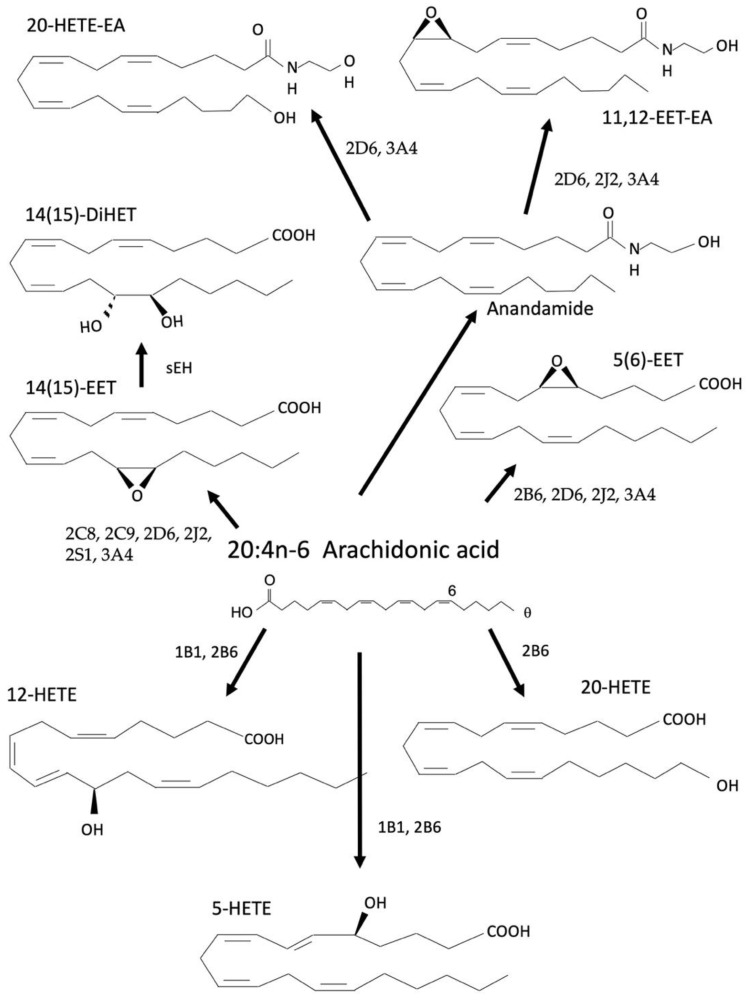
Metabolism of arachidonic acid by CYPs produces multiple products such as the EETs that are subsequently metabolized by sEH to a corresponding DiHET. Other metabolites include but are not limited to 19-HETE, 9,10-EET, 11,12-EET, and the subsequent sEH DiHET products.

Arachidonic acid (AA; 20:4) is an n-6 PUFA comprised of a 20-carbon chain with four double bonds [96]. While AA can be synthesized from LA, it is more commonly consumed through the diet similarly to LA [97]. Primary sources include meats such as beef, poultry, pork, and some fish [96,97]. AA is metabolized by CYP enzymes to form primarily the epoxyeicosatrienoic acids (EETs) that are subsequently metabolized to the dihydroxyeicosatrienoic acids (DiHETs) by sEHs. Furthermore, hydroxyeicosatetraenoic acid (HETEs) are formed from LOX and CYP metabolism [98] (Figure 3).

AA can be directly metabolized to HETEs by CYPs without the intermediate HpETEs similar to the HODEs from LA [99]. No studies currently demonstrate that AA is metabolized to HpETEs by the CYPs, only the direct synthesis of HETEs [99]. The most prominent of the hepatic CYPs, CYP3A4 produces multiple HETEs and EETs. CYP3A4 oxygenates AA to 13-HETE, 10-HETE, and 7-HETE [75]. The epoxides formed from CYP3A show stability, but are also metabolized by sEH to diols [100]. Inhibition assays suggest that a Cyp3a-mediated arachidonic acid EET is in part responsible for relaxation of arterial endothelium [101].

HETEs can be further metabolized by dehydrogenases to the oxoicosatetraenoic acids (oxoETEs) (not shown in Figure 3) [102]. While both HODEs produced through CYPs have a respective oxoODE, only three of the seven HETEs are further metabolized to oxoETE, 5-, 12-, and 15-oxoETE [1]. CYPs can also metabolize AA to the EETs at any of the double bond positions. Each CYP preferentially produces one or two regioisomers while the other regioisomers are produced at lower levels [103]. For example, rat CYP2B’s primarily produces 11,12-EET in the liver but also produces moderate amounts of 8,9-EET and 14,15-EET [104]. Similarly to the epoxides generated from LA, soluble epoxide hydrolases (sEHs) can further metabolize EETs to DiHETs [105], although their role in signaling pathways is not as well established as their predecessors [105].

The HETEs are generally regarded as inflammatory, with many of them contributing to vasoconstriction and inflammatory pathways. For example, 5-HETE has been shown to induce neutrophil migration leading to airway constriction [106], which is accompanied by an increase in intracellular calcium as a result of neutrophil activation [99]. Other HETEs also contribute to vasoconstriction such as 15-HETE through the PGH2/TXA2 receptors resulting in increased pulmonary artery tension [107] and 20-HETE that constricts vascular smooth muscle through blocking activity of the calcium-activated potassium channel and enhancing the activity of voltage-gated L-type calcium channels [108]. However, 20-HETE, a ligand of GPR75, that has been well studied for its pro-inflammatory and proliferative activity is primarily produced by CYP4A and CYP4F members [109]; not the CYP1-3 family members. See Table 4 for a summary of AA-derived oxylipin actions.

In contrast, some HETEs participate in anti-inflammatory responses. For example, 5-HETE has been shown to activate Nrf2 [110], which is an important transcription factor that regulates anti-oxidant responses [111]. This points to 5-HETE as not only being inflammatory but also having a secondary anti-inflammatory role in signaling for protection against the oxidative stress produced during the initial inflammatory reactions.

Platelet aggregation is enhanced by 12-HETE, a ligand of GPR31 [112], while exposure to 19-HETE, produced by CYP2E1, results in activation of the prostacyclin (IP) receptor resulting in reduced platelet aggregation [98]. The activation of the IP receptor by 19-HETE also reduces vascular constriction [98], which is in direct opposition of the inflammatory activity generated by most HETEs. Mid-chain HETEs were decreased in mice over-expressing endothelial sEH, but these mice also experienced decreased coronary reactive hyperemia [113], which indicates the role of HETEs in inflammatory events in the cardiovascular system may be more complicated than previous studies demonstrate. These diverging roles in inflammation show the diversity of responses elicited by these oxylipins.

Some of the HETEs are less well-studied, so little is known about their activity. 9-HETE, for example, acts as a marker for oxidative stress and is elevated in patients with coronary artery disease [114], but little is known about whether it contributes to a mechanism responsible for the disease. Another HETE that has been left largely uninvestigated is 18-HETE. One study found it increases vasodilation in rabbit kidney [115]; however, few other studies have shown biological activity or divulged a mechanism.

The EETs work through a variety of different mechanisms, and unlike the HETEs they are generally regarded as anti-inflammatory although they may also demonstrate pro-inflammatory responses. Several of the EETs signal the same receptors, for example 8,9-, 11,12-, and 14,15-EET all activate the JNK/c-Jun pathway to stimulate pulmonary artery endothelial cells proliferation and angiogenesis [116]. The JNK pathway is also associated with several diseases, including obesity, steatosis, atherosclerosis, and others [8,117].

These oxylipins also act as potentially anti-inflammatory signaling molecules that decrease epithelial sodium channel activity and reduce sodium reabsorption [118]. This impairment of sodium reabsorption channels has been shown to contribute to a decrease in blood pressure [119]. 14,15-EET also suppresses mitochondrial apoptosis during ischemia-reperfusion injury through the PI3K/AKT/CREB/Bcl-2 signaling pathway [120], which could possibly reduce the rate of apoptosis seen in muscle cells in response to metabolic diseases such as dyslipidemia [121]. 5,6-EET does not signal through the previously mentioned pathways and instead functions to suppress cardiomyocyte shortening [122], which may be a result of its action as an inhibitor of T-type calcium channels that contribute to vascular tone [123]. 11,12- and 14,15-EET levels were increased in endothelial CYP2J2-overexpressing mice, and these mice had improved coronary reactive hyperemia [83].

Murine Cyp2b19 and rat CYP2B12 are primarily found in keratinocytes and important in 14,15-EET formation, a key factor in epithelial cornification [124,125,126]. Interestingly, recent data provides an association between Cyp2b repression (also Cyp2j/4a/2c) and development of NAFLD during a high-fat diet, putatively due to a lack of arachidonic acid expoxygenase activity [8,44,127].

The EETs are also considered protective in the brain because of their anti-inflammatory and anti-thrombotic activities [128,129,130]. Furthermore, disruption of EET metabolism altered behavior in sEH knockout mice, but not completely in an expected manner, as these mice showed improved motor skills but reduced learning capacity for spatial memory [131].

AA and ethanolamine undergo enzymatic reactions to yield an n-6 endocannabinoid, anandamide (AEA), although this synthesis pathway requires substantial amounts of free AA [132]. AEA can then be metabolized by CYPs to yield several AEA-derived oxylipins with similar sites of metabolism to the AA derivatives. Many of these AEA-derived oxylipins are not well studied, but several have been shown to activate the cannabinoid (CB) receptors. For example, 5,6-EET-EA is a potent activator of both CB1 and CB2 [133], while 11,12-EET-EA is only an agonist of CB2 [134]. 20-HETE-EA is also an agonist of the CBs, but it has a very low binding affinity compared to 5,6- and 11,12-EET-EA [135]. 5,6-, 8,9-, and 14,15-EET-EA can activate a different receptor called the GPR119 receptor, which results in an increase in intracellular cAMP, and a reduction in the innate immune response [136,137]. CYP3A4 is considered the key CYP in anandamide metabolism with CYP2D6 and CYP4F2 playing smaller roles [133,138]. This provides further evidence that loss of Cyp3a activity may perturb endocannabinoid action, alter immune response and perturb mood.

**Table 4:** Metabolism of arachidonic acid produces several oxylipins with a diverse set of putative functions.

Oxylipin	CAS Number	CYPs/Enzymes	References	Effects	References
5-HETE	330796-62-8	1B1, 2B6	[139,140]	Activates Nrf2 Stimulate neutrophils to increase intracellular calcium Induce airway contraction through induction of neutrophil migration	[99,106,110]
9-HETE	79495-85-5	2B6	[140]	Marker for oxidative stress	[114]
12-HETE	71030-37-0	1B1, 2B6	[8,139,140]	Contributes to platelet aggregation	[112]
15-HETE	71030-36-9	1B1	[139]	Can increase pulmonary artery tension through PGH2/TXA2 receptors	[107]
18-HETE	133268-58-3	2E1	[141]	Induces vasodilation in rabbit kidney	[115]
19-HETE	79551-85-2	2E1, 2U1	[141,142]	Activates the prostacyclin (IP) receptor, inhibiting platelet aggregation and reducing vascular constriction	[98]
20-HETE	79551-86-3	2U1	[142]	Acts as a participant in tubuloglomerular feedback response in the kidney Promotes salt excretion through inhibition of the Na^+^-K^+^-ATPase and Na^+^-K^+^-2Cl^−^ cotransporters Constricts vascular smooth muscle through blocking activity of the calcium-activated potassium channel and enhancing the activity of voltage-gated L-type calcium channels	[108,143,144]
5,6-EET	81246-84-6	2B6, 2D6, 2J2, 3A4	[8,138,145,146]	Can suppress cardiomyocyte shortening Inhibits T-type calcium channels which may contribute to vascular tone	[122,123]
8,9-EET	184488-44-6	1A2, 2B6, 2C9, 2D6, 2J2 3A4	[8,138,140,145,146,147,148]	Inhibits B-cell proliferation and survival, possibly through inhibition of Nf-κB Stimulates pulmonary artery endothelial cells proliferation and angiogenesis through the JNK/c-Jun pathway Decreases epithelial Na^+^ channel activity to reduce sodium reabsorption	[116,118,149]
11,12-EET	200960-01-6	2C8, 2C9, 2D6, 2J2, 2S1, 3A4, 2B	[104,138,145,148,150]	Stimulates pulmonary artery endothelial cells proliferation and angiogenesis through the JNK/c-Jun pathway Decreases epithelial Na^+^ channel activity to reduce sodium reabsorption Activates the α and β_1_ subunits of mitochondrial BK channels to promote pulmonary vasoconstriction Increased following eschemia in endothelial CYP2J2-overexpressing mice	[83,116,118,151]
14,15-EET	197508-62-6	2C8, 2C9, 2D6, 2J2, 2S1, 3A4	[138,145,148,150]	Stimulates pulmonary artery endothelial cells proliferation and angiogenesis through the JNK/c-Jun pathway Decreases epithelial Na^+^ channel activity to reduce sodium reabsorption Increased following eschemia in endothelial CYP2J2-overexpressing mice Suppresses mitochondrial apoptosis through the PI3K/AKT/CREB/Bcl-2 signaling pathway in ischemia–reperfusion injury	[83,116,118,120]
5,6-EET-EA *	N/A	2D6, 2J2, 3A4	[152,153]	Potent agonist of CB1 and CB2 Weak agonist of GPR119 receptor	[133,137]
8,9-EET-EA *	N/A	2D6, 2J2, 3A4	[152,153]	Agonist of GPR119 receptor	[137]
11,12-EET-EA *	N/A	2D6, 2J2, 3A4	[152,153]	High-affinity agonist of CB2	[134]
14,15-EET-EA *	N/A	2D6, 2J2, 3A4	[152,153]	Weak agonist of GPR119 receptor	[137]
19-HETE-EA *	N/A	2D6, 3A4	[152,153]	Unknown	
20-HETE-EA *	942069-11-6	2D6, 3A4	[34,152,153]	Low affinity binding of CB	[135]

* denotes an oxylipin derived from anadamide (AEA).

There is significant competition between linoleic acid and arachidonic acid oxylipins during inflammation. Under normal conditions the metabolites of linoleic acid dominate and both EpOMEs and DiHOMEs are measurable probably because of the higher substrate concentration of LA. Upon inflammation the arachidonic acid metabolites dominate; most produced by CYP2J and CYP2C members. EETs are not highly stable and therefore sometimes they are not found or measured at low levels. Instead the DiHETs are primarily measured, which are more likely pro-inflammatory similar to the linoleic acid oxylipins; and unlike the anti-inflammatory EETs that provide protection from lung or cardiac injuries following the initial influx of CYP-derived oxylipins. Therefore, inhibition of sEH may provide benefits for inflammatory resolution [18]. Interestingly, the EPA and DHA derived oxylipins did not change during inflammatory resolution [18]. Therefore, competition for CYP metabolism by other PUFAs such as the n-3’s through an improved diet could also inhibit metabolism of AA to pro-inflammatory oxylipins and improve outcomes.

### 2.3. α–Linolenic Acid Metabolism

- ALA is metabolized by the CYPs into a number of distinct oxylipins including the EpODEs and HOTrEs
- Less is known about the individual CYPs responsible for metabolism of ALA
- There are several ALA-derived oxylipins about which little is known or little confirmation of its activity.

Alpha-linolenic acid (ALA; 18:3) is an n-3 PUFA comprised of an 18-carbon chain with three double bonds [154]. Like LA, ALA is an essential fatty acid, meaning it cannot be synthesized by humans and must be consumed through diet [154]. It is found in several plant-based oils as well as nuts and some leafy vegetables [155].

**Figure 4:**
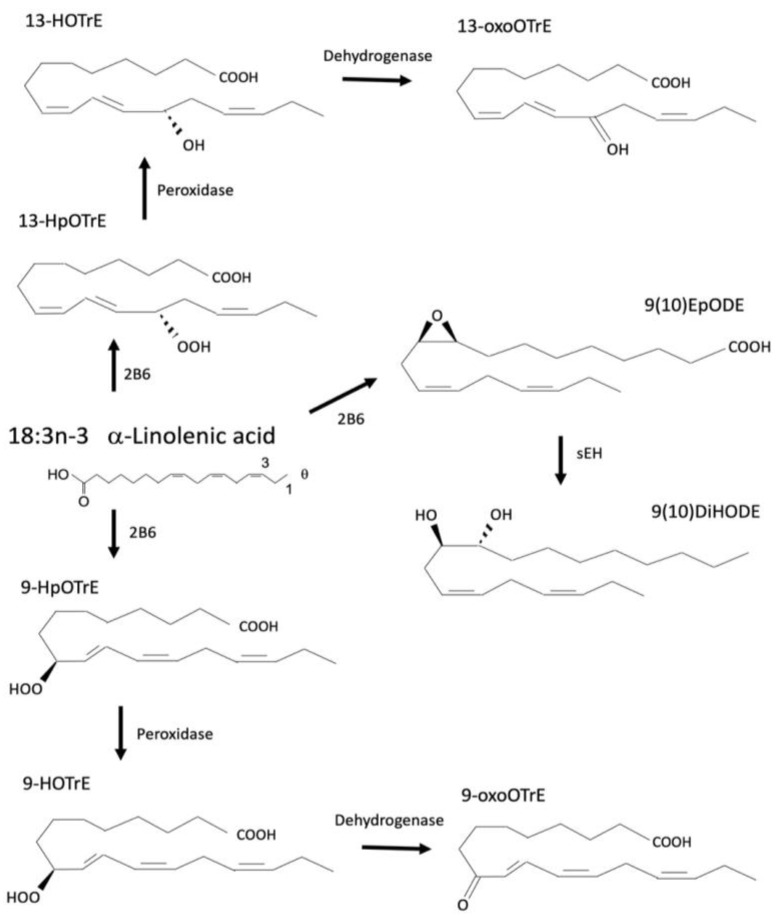
Metabolism of a-linolenic acid a produces multiple products such as the EpODEs that are subsequently metabolized by sEH to a corresponding DiHODEs. Other metabolites include but are not limited to HOTrEs and HpOTrEs. Recent research with CYP2B6 provides a preferred metabolism of PUFAs, especially ALA, in the 9 or 13 positions.

ALA can be converted to eicosapentaenoic acid (EPA) or docosahexaenoic acid (DHA), although this conversion seems to be limited in humans [121]. ALA is metabolized by CYPs to form epoxy-octadecadienoic acids (EpODEs) or potentially hydroperoxy-octadecatrienoic acids (HpOTrEs), which like their LA derivatives can be further metabolized by sEH or peroxidases to dihydroxy-octadecadienoic acids (DiHODEs) or hydroxy-octadecatrienoic acids (HOTrEs), respectively [1]. HOTrEs can then be metabolized by dehydrogenases to oxo-octadecatrienoic acid (oxoOTrEs) [1] (Figure 4). The CYPs responsible for ALA metabolism have not been well established. HOTrE and HpOTrE metabolism is carried out by LOX, but a recent paper shows that ALA is a preferred PUFA substrate for CYP2B6 with oxidative preference at the 9- and 13- positions. 9-HOTrE also activates PPARα [8].

Very little is known about the effects of ALA-derived oxylipins (Table 5). While concentrations of these oxylipins have been associated with a variety of conditions, little is known about whether these oxylipins are involved in the mechanism of these effects or diseases. For example, DiHODE concentrations in hyperlipidemic men were decreased compared to normolipidemic men [156], but no follow up on this study has been completed. EpODE concentrations have been shown to be significantly increased in male rats upon treatment with ibuprofen [157]; however, the cause or effects of this change in oxylipin profile have yet to be investigated. Several of these oxylipins have been associated with pregnancy and gestation. 9,10- and 15,16-DiHODE have been found at higher concentrations in the corpus lutea in pregnant cattle [158], and lower levels of 9,10-DiHODE have been associated with an increase in preterm delivery prior to 34 weeks [159], but the implications of this have not been determined.

13-HpOTrE and 13-HOTrE both inactivate the NLRP3 inflammasome [160] that is responsible for the release of the pro-inflammatory cytokines IL-1β and IL-18 [161]. This suggests they play a role in reducing inflammation. Both 9- and 13-HOTrE have been shown to reduce lipid droplet accumulation in 3T3-L1 adipocytes [162], but a mechanism for this has not been determined. 13-HOTrE has recently been shown to increase gene expression of the Sterol regulatory-element binding factors (SREBFs) as well as fatty acid synthase (FASN) in murine skeletal muscle cells [163], which may indicate a role in signaling for lipid metabolism and biosynthesis. Further research into ALA-derived oxylipins is needed to enhance understanding of their involvement in these effects, as the current literature is lacking.

**Table 5:** Metabolism of α-linolenic acid produces several oxylipins with a diverse set of putative functions.

Oxylipin	CAS Number	CYPs/Enzymes	References	Effects	References
9,10-EpODE	N/A	2B6	[8]	Significantly increased in male rats treated with ibuprofen	[157]
12,13-EpODE	N/A	unknown		Significantly increased in male rats treated with ibuprofen	[157]
15,16-EpODE	N/A	2B6	[8]	Significantly increased in male rats treated with ibuprofen	[157]
9,10-DiHODE	N/A	sEH	[1]	Found at decreased concentrations in hyperlipidemic men vs. normolipidemic men Lower levels in pregnant women have been associated with increase in preterm delivery before 34 weeks Higher in corpus lutea in pregnant cattle	[156,158,159]
12,13-DiHODE	N/A	sEH	[1]	Found at decreased concentrations in hyperlipidemic men vs. normolipidemic men	[156]
15,16-DiHODE	N/A	sEH	[1]	Found at decreased concentrations in hyperlipidemic men vs. normolipidemic men Higher in corpus lutea in pregnant cattle	[156,158]
9-HpOTrE	111004-08-1	2B6	[8]	unknown	
13-HpOTrE	67597-26-6	2B6	[8]	Inactivate the NLRP3 inflammasome	[160]
9-HOTrE	89886-42-0	2B6	[8]	Increased concentrations in patients with perioperative dry eye syndrome Reduced lipid droplet accumulation in 3T3-L1 adipocytes	[162,164]
13-HOTrE	87984-82-5	2B6	[8]	Inactivate the NLRP3 inflammasome Increased concentrations in patients with perioperative dry eye syndrome Reduced lipid droplet accumulation in 3T3-L1 adipocytes Increase SREBF1, SREBF2, and FASN gene expression in C2C12 murine skeletal muscle cells	[160,162,163,164]
9-oxoOTrE	125559-74-2	dehydrogenase	[1]	Shows antimicrobial activity in plants	[165]
13-oxoOTrE	N/A	dehydrogenase	[1]	unknown	

### 2.4. Eicosapentaenoic and Docosahexaenoic Acid Metabolism

- DHA and EPA are metabolized by CYPs, including CYP2C, CYP2J, and CYP3A subfamily members. EPA is metabolized into the HEPEs, epoxidated to the EpETEs and in turn the diols, DiHETEs (DHETEs) by sEH; DHA is metabolized to the epoxidated EpDPAs and in turn the DiHDPAs by sEH.
- DHA and EPA often function as anti-inflammatory and perceived as beneficial; however there are examples of negative effects of their oxylipins, especially DHA.
- Some of DHA and EPA’s beneficial effects are probably due to competitive inhibition of AA metabolism.

**Figure 5:**
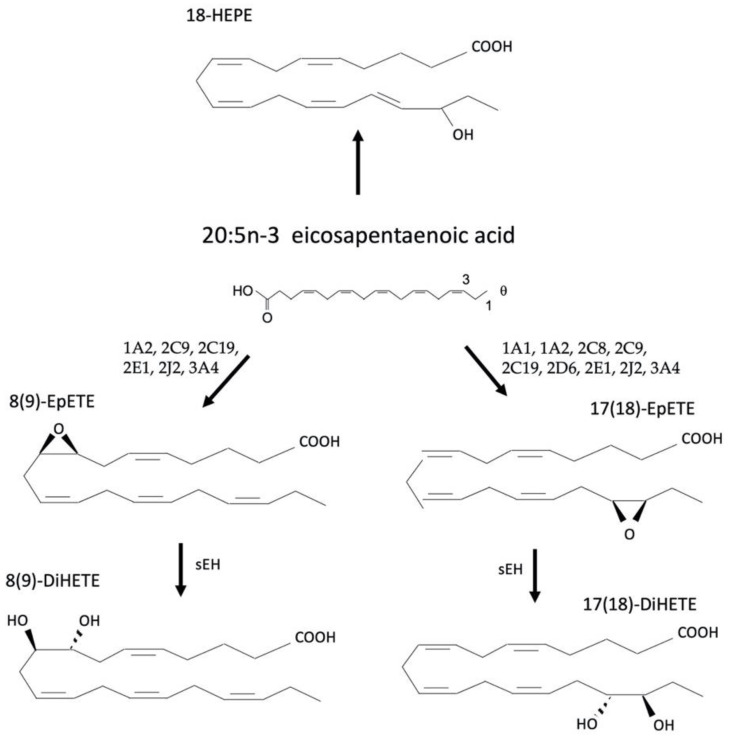
Metabolism of eicosapentaenoic acid (EPA) a produces multiple products such as the EpETEs that are subsequently metabolized by sEH to a corresponding DiHETEs and the HEPEs.

**Figure 6:**
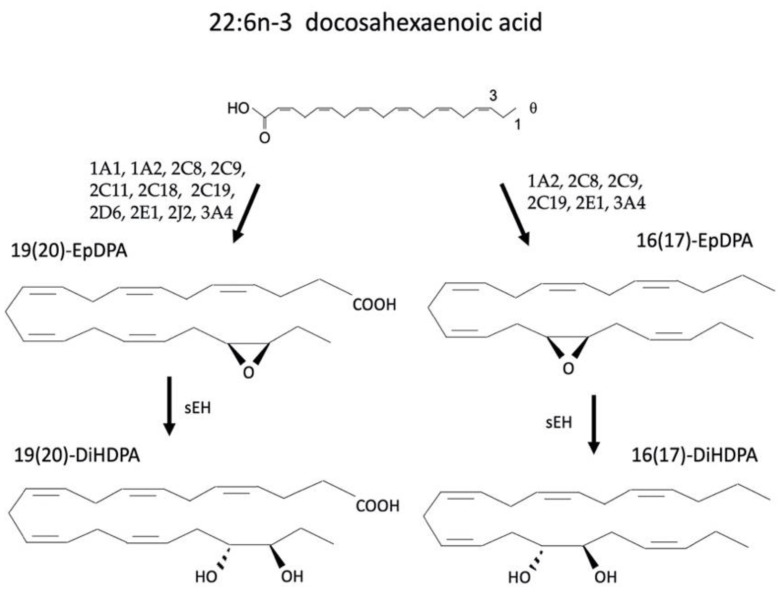
Metabolism of eicosapentaenoic acid (EPA) a produces multiple products such as the EpETEs that are subsequently metabolized by sEH to a corresponding DiHETEs and the HEPEs.

Eicosapentaenoic acid (EPA; 20:5) and docosahexaenoic acid (DHA; 22:6) are n-3 PUFAs comprised of 20 and 22 carbons, respectively. EPA and DHA can be synthesized from ALA, an essential PUFA; however, they are more commonly consumed through the diet such as salmon, trout, tuna, cod, oysters, flaxseed, walnuts, and soybeans. EPA is primarily metabolized by CYP enzymes to form the epoxides EpETEs such as 5,6-EpETE, 8,9-EpETE and others. These are then metabolized by sEH to produce the DiHETEs. Other CYP-derived oxylipins from EPA include the HEPEs such as 18-HEPE, 19-HEPE, and 20-HEPE (Figure 5). DHA is primarily metabolized across its double bonds to the epoxidated EpDPAs such as 13,14-EpDPA or 17,18-EpDPA with subsequent sEH-mediated hydrolysis to their respective diols, 13,14-DiHPDA or 17,18-DiHPDA (Figure 6).

The serum levels of the n-3 PUFAs mimic the consumption patterns of n-3 PUFAs and this is also true for their oxylipins. In turn, the many anti-inflammatory and anti-proliferative effects of oxylipins are provided by eating better diets [166]. Diets high in EPA and DHA increased EPA and DHA-derived oxylipins [167,168], and decreased AA-derived oxylipins, possibly through direct inhibition of CYP-mediated metabolism. A recent manuscript evaluated the production of 17,18-EpETE, an EPA oxylipin, from each of the murine CYPs. 17,18-EpETE was produced from EPA by Cyp1a, 2a, 2b, 2c, 2j, 3a, 4a, 4f, 26, and 46 members with Cyp4a12a > 1a2 > 4f18 > 4a12b > 2c50 > 2c38 > 2b10 in production of this oxylipin [169]. Further metabolite analysis showed that Cyp1a2 produced 18-HEPE and 19-HEPE, Cyp2c50 produced a large number of EPA oxylipins, and Cyp4a12a and Cyp4f18 produced 18-HEPE, 19-HEPE, and 20-HEPE (Cyp4a12a only). Human CYP1A2 produced similar metabolites as murine Cyp1a2. Human CYP4, CYP1A, and CYP2C members are typically considered important in the metabolism of n-3 fatty acids [170,171].

Many investigators believe that the association between n-3 PUFAs and better health outcomes is caused by the formation of the n-3 oxylipins [167,168]. For example, 19,20-EpDPA produced from DHA and to a lesser extent 14,15-EET produced from AA are CYP-mediated oxylipins that lower blood pressure caused by angiotensin II [171]. Omega-3 PUFAs are also anti-obesogenic and have anti-cancer properties probably because of their anti-inflammatory and anti-oxidant effects [172]. The EPA and DHA oxylipins 17,18-EpETE and 19,20-EpDPE, respectively, activate GRP120 and AMPKa and in turn increase brown adipose tissue thermogenesis and increase the beiging of white adipose tissue [173]. Both of these increase metabolism and can decrease obesity. DHA oxylipins are associated with better cardiovascular outcomes, reduced cardio-toxicity caused by LPS, reduced lung cancer colonies, reduced metastasis, lower blood pressure in the obese, and improved fatty liver indices (Table 6 and Table 7) [169,170,174,175,176,177,178].

However, negative outcomes occur as well. For example, diets high in EPA, DHA, EPA + DHA, or none of the n-3’s were provided and several inflammatory biomarkers were measured. EPA produced positive health outcome associations between oxylipins, IL-6, and bronchoalveolar lavage fluid as did the EPA + DHA group. However, DHA alone increased CYP and LOX derived oxylipins as well as increased IL-6 and bronchoalveolar lavage. Therefore, several DHA oxylipins may be pro-inflammatory in the lung [168]. Other negative effects of n-3 oxylipins include association with increased seasonal depression, liver fibrosis, and soybean oil induced obesity [2,187,188].

**Table 7:** Metabolism of docosahexaenoic acid produces several oxylipins with a diverse set of putative functions.

Oxylipin	CAS Number	CYPs/Enzymes	References	Effects	References
7,8-EpDPA	895127-66-9	1A2, 2C9, 2C19, 2J2, 3A4	[179]	Increased in hemodialysis patients	[189]
10,11-EpDPA	895127-65-8	1A2, 2C8, 2C9, 2C19, 2J2, 3A4	[179]	Increased in hemodialysis patients	[189]
13,14-EpDPA	895127-64-7	1A2, 2C8, 2C9, 2C19, 2J2, 3A4	[179]	Activates large-conductance calcium-activated potassium in smooth muscle in coronary arteries Increased in hemodialysis patients	[189,190]
16,17-EpDPA	155073-46-4	1A2, 2C8, 2C9, 2C19, 2E1, 3A4	[179]	Inhibits VEGF-induced angiogenesis and significantly reduces metastasis Increased in hemodialysis patients	[177,189]
19,20-EpDPA	N/A	1A1, 1A2, 2C8, 2C9, 2C11, 2C18, 2C19, 2D6, 2E1, 2J2, 3A4	[179,191]	Potent vasodilators in microcirculatory vessels Protects cardiac cells against lipopolysaccharide-induced toxicity through activation of Sirtuin 1 which positively regulates LXR Inhibits VEGF-induced angiogenesis Increased in hemodialysis patients Increase brown adipose tissue thermogenesis	[173,177,178,189,192]
7,8-DiHDPA	168111-93-1	sEH		Lower concentrations found in the brains of G-protein coupled receptor 39-knock out mice fed a high fat diet	[193]
10,11-DiHDPA	1345275-22-0	sEH		unknown	
13,14-DiHDPA	1345275-24-2	sEH		Negatively correlated with atherosclerotic cardiovascular disease risk	[176]
16,17-DiHDPA	1345275-27-5	sEH		Increased concentrations found in patients with seasonal depression during winter months	[188]
19,20-DiHDPA	N/A	sEH		Reduced concentrations found in mice treated with fenofibrate, a PPARα activator	[194]

Neovascularization of the retina is a cause of blindness. Omega-3 fatty acids can reduce the vascularization; however, some CYP-mediated oxylipins promote ocular pathological angiogenesis. In the retina, CYP2C metabolizes AA to 14,15-EET and DHA to 19,20-EpDPA that are subsequently metabolized to 14,15-DiHET and 19,20-DiHDPA by sEH. Inhibition of CYP2C by the CYP2C8 inhibitor, montelukast significantly reduces pathological blood vessel formation. Inhibition of sEH increases ocular neovascularization indicating the the 14,15-EET and 19,20-EpDPA metabolites are responsible for inducing neovascularization and promoting the pathological blindness. Furthermore, direct treatment with 19,20-EpDPA overcame CYP2C inhibition leading to neovascularization. Thus, specific AA and DHA epoxy—oxylipins are critical in ocular neovascular disease progression and blindness [195].

Exercise increases serum oxylipin levels from fasted athletes of several PUFAs, especially AA, DHA, and EPA from CYPs. This also includes the LA-derived HODEs that could be produced from LOX or CYP activity. Interestingly, providing carbohydrates immediately after the exercise reduced oxylipin production with the reduction of CYP-mediated oxylipins most prominent [196]. The mechanism is not known, but may involve the influx of insulin and in turn the repression of lipase activity leading to reduced substrate levels. The benefits of post-workout carbohydrates may be reduced inflammation from a reduction in AA-based oxylipins such as the HETEs and an increase in EETs caused by a drop in sEH activity. Furthermore, few pro-resolvin mediators were not measured immediately post-exercise [196]. The mechanism for CYP activity repression is not known, but hypotheses include reduced insulin or fatty acid mediated induction [44,58,197]. However, not all PUFAs are equal as most induce CYP activity such as LA [58,198,199,200,201], which was released early into the serum during this exercise study. However, other PUFAs are inhibitors of CYP induction. For example, DHA directly inhibits CAR-regulated CYP induction [202].

Interestingly, the endocannabinoid derivatives of the n-3 oxylipins often have stronger physiological effects than their precursors [166]. A recent review summarizes their anti-inflammatory, anti-cancer, anti-obesity, energy sensing capabilities, as well as role in food intake [172]. Other studies have demonstrated significant anti-inflammatory properties, anti-cancer, and anti-anxiety or anti-depression [203]. However, there has been much less study of the n-3 endocannabinoids and therefore more research is necessary [204,205,206].

## 3. Discussion—Potential Interactions

Several different CYPs are key contributors to PUFA metabolism with CYP4A and 4F playing prominent roles in omega-oxidation of AA. However, many other CYPs are also involved in PUFA metabolism and the formation of oxylipins. Several of these are in the CYP familes 1–3; the same families involved in detoxification of endo- and xenobiotics. These detoxification CYPs are often highly inducible through the activation of xenosensors such as AhR, CAR, PXR, and others [19]. PPARs can also induce several CYP subfamilies; most prominently the CYP4A subfamily important in omega hydroxylation of fatty acids [207]. The CYP4A subfamily does not fit under the detoxification CYPs and part of this review, but they are inducible and important in PUFA metabolism, especially AA.

Chemicals that activate AhR, CAR, and PXR are likely to increase oxylipin formation. A great example is dioxin, a crucial inducer of CYP1A members that also significantly increases PUFA metabolism to oxylipins [208]. Quercetin activates CAR, increases omega-oxidation of multiple PUFAs and reduces serum lipids [209]. Overall, these changes most likely lead to downstream effects that probably vary based on the diet. For example, a diet rich in n-6 fatty acids would certainly be more pro-inflammatory that an n-3 diet. Thus, oxylipin metabolism and effects are dependent on chemical exposure and diet.

Serum oxylipins such as 15-HETE, 12-HEPE, 17-hDHA, and 5,6-DHET were increased by airborne particular matter. The CYPs responsible for these products under these conditions are not known. As most of these are considered pro-inflammatory, specific oxylipins may provide information about the health of our environment; diet, chemical exposure, etc. [210]. PM and the PAHs that may be present within them are likely CYP inducers and in turn support an unhealthy, pro-inflammatory internal environment that is more prone to obesity, diabetes, and cancer.

DHA acts as an inhibitor of CYP2B6, CYP2C8, CYP3A4 and other CYPs as do several other PUFAs with EC50s in the low micromolar range (1–10 μM). EPA, DHA, and AA have greater inhibitory capacity than LA and ALA for most CYPs [36,183,211]. With a EC50 in the low micromolar range, most inhibition would occur directly after a meal, after pharmacological treatment with a PUFA, in the presence of high amounts of free fatty acids such as in a steatotic condition, or with a mixture of other PUFAs. DHA inhibits AA oxylipin formation and has the benefit of being a n-3 PUFA with reasonably strong inhibition of most CYPs including CYP3A4 [36,183]. A diet rich in n-3 PUFAs may also provide reduced inflammation through competition for CYP metabolism and ultimately inhibition.

DHA has been used to inhibit the CYP3A-mediated metabolism and increase the retention of some drugs, including midazolam and cyclosporin [212,213]. DHA also represses the translocation of CAR, a key nuclear receptor involved in the induction of CYP2B and to a lesser extent CYP3A enzymes. This may provide another mechanism by which DHA can repress CYP activity [202]. Taken together, DHA and potentially other PUFAs can cause drug-drug or diet-drug interactions and potentially used to beneficial effects [29,36,202].

Drugs can also be used to inhibit adverse effects from CYP-mediated PUFA metabolism. Inhibition of CYP2C metabolism of DHA and AA can have pharmacological effects and improve pathological neovascularization [195]. Inhibition of CYP3A4 and perhaps CYP2J members by ketoconazole reduces the production of HODES and potentially 12,13-DiHOME responsible for dental pain [16,214]. A diet high in n-3 fats may provide health benefits alone and/or be used to potentiate the effects of some drugs (see above).

Several genetic, biochemical, and environmental effects can effect the abundance and type of oxylipins produced. These include (1) diet, especially PUFA type in the diet. (2) Liver steatosis or steatohepatitis, which leads to induction or repression of CYP expression, respectively. (3) Chemical exposure as several environmental chemicals such as pesticides, plasticizers, fire retardants, and many more induce CYPs through AhR, CAR, PXR, etc., and in turn may increase oxylipin production; another potential mechanism by which environmental chemicals could cause oxidative stress or inflammation. (4) Several phamaceuticals are CYP modulators through the same mechanisms mentioned above for environmental chemicals and may cause drug-drug interactions because of these effects (5) Hormones and bile acids may also alter CYP expression through PXR, CAR, or FXR. (6) Last, polymorphisms such as those in CYP2B6 or CYP2D6 disrupt endocannaboid oxylipin production [34]. Taken together, oxylipins are often present in the serum at ratios similar to the diet and produced by a variety of CYPs whose expression may not be stable. Therefore, oxylipin levels are contingent on our diet and CYP activity, which are altered by a variety of environmental factors.

## 4. Conclusions

The production of oxylipins occurs through multiple pathways, is inducible, and can have both positive and negative consequences. Our understanding of the role of CYPs in the production of oxylipins is growing, but the role of specific CYPs is still understudied. Our knowledge of the individual CYP-derived oxylipins is also growing; however, there are many oxylipins that have not been investigated or mechanistic studies are lacking. Further study of the function of CYP-derived oxylipins will increase our understanding of oxylipin signaling and the interaction between our diet, environment, and sex. Understanding the role of the specific CYPs will help us understand and provide mechanisms by which modulation of CYPs will alter oxylipin production and effect. More importantly, dietary or pharmacological interventions may be available to enhance the desired effects and inhibit the negative effects of oxylipins. Overall, our diet, environment, age, pharmaceutical treatments, etc., are likely to affect our oxylipin production, their ratios, and their effects; both negative and beneficial.

## Figures and Tables

**Table 1:** Orthologous detoxification CYP subfamilies between human, rat, and mouse.

CYP Subfamily	Human	Mouse	Rat
CYP1A	CYP1A1, 1A2	Cyp1a1, 1a2	Cyp1a1, 1a2
CYP1B	CYP1B1	Cyp1b1	Cyp1b1
CYP2A	CYP2A6, 2A7, 2A13	Cyp2a4, 2a5, 2a12, 2a22	Cyp2a1, 2a2, 2a3
CYP2B	CYP2B6	Cyp2b9, 2b10, 2b13, 2b19, 2b23	Cyp2b1, 2b2, 2b3, 2b12, 2b15, 2b21
CYP2C	CYP2C8, 2C9, 2C18, 2C19	Cyp2c29, 2c37, 2c38, 2c39, 2c40, 2c44, 2c50, 2c54, 2c55, 2c65, 2c66, 2c67, 2c68, 2c69, 2c70	Cyp2c6, 2c7, 2c11, 2c12, 2c13, 2c22, 2c23, 2c24, 2c78, 2c80
CYP2D	CYP2D6	Cyp2d9, 2d10, 2d11, 2d12, 2d13, 2d22, 2d26, 2d34, 2d40	Cyp2d1, 2d2, 2d3, 2d4, 2d5
CYP2E	CYP2E1	Cyp2e1	Cyp2e1
CYP2J	CYP2J2	Cyp2j5, 2j6, 2j7, 2j8, 2j9, 2j11, 2j12, 2j13	Cyp2j3, 2j4, 2j10, 2j13, 2j16
CYP2S	CYP2S1	Cyp2s1	Cyp2s1
CYP2U	CYP2U1	Cyp2u1	Cyp2u1
CYP3A	CYP3A4, 3A5, 3A7, 3A43	Cyp3a11, 3a13, 3a16, 3a25, 3a41, 3a44, 3a57, 3a59	Cyp3a1, 3a2, 3a9, 3a18, 3a23, 3a62, 3a73

**Table 2:** Primary CYP-mediated oxylipins produced from different PUFAs.

PUFA	Abbreviation	PUFA Type	Oxylipins Produced by CYPs
Linoleic acid	LA	n-6	EpOME, DiHOME, HpODE, HODE, oxoODE
Arachidonic acid	AA	n-6	HETE, oxoETE, EET, DiHET
alpha-linolenic acid	ALA	n-3	EpODE, DiHODE, HpOTrE, HOTrE, oxoOTrE
Eicosapentaenoic acid	EPA	n-3	EpETE, DiHETE, HEPE
Docosahexaenoic acid	DHA	n-3	EpDPA, DiHDPA

Epoxyoctadecamonoenoic acid (EpOME), dihydroxyoctadecenoic acid (DiHOME), hydroperoxy-octadecadienoic acid (HpODE), hydroxy-octadecadienoic acid (HODEs), oxo-octadecadienoic acid (oxoODE), hydroxyeicosatetraenoic acid (HETE), oxoicosatetraenoic acid (oxoETE), epoxyeicosatrienoic acid (EET), dihydroxyeicosatrienoic acids (DiHETs), epoxy-octadecadienoic acid (EpODE), dihydroxy-octadecadienoic acid (DiHODE), hydroperoxy-octadecatrienoic acid (HpOTrE), hydroxy-octadecatrienoic acid (HOTrE), oxo-octadecatrienoic acid (oxoOTrE), epoxy-eicosatetraenoic acid (EpETE), dihydroxy-eicosatetraenoic acid (DiHETE), hydroxyicosapentaenoic acid (HEPE), epoxy-docosapentaenoic acid (EpDPA), dihydroxy-docosapentaenoic acid (DiHDPA).

**Table 6:** Metabolism of eicosapentaenoic acid produces several oxylipins with a diverse set of putative functions.

Oxylipin	CAS Number	CYPs/Enzymes	References	Effects	References
5,6-EpETE	N/A	2C9, 2J2	[179]	unknown	
8,9-EpETE	851378-93-3	1A2, 2C9, 2C19, 2E1, 2J2, 3A4	[179]	unknown	
11,12-EpETE	504435-15-8	1A2, 2C8, 2C9, 2C19, 2E1, 2J2, 3A4	[179]	Higher levels are associated with lower blood pressure in obese children	[180]
14,15-EpETE	131339-24-7	1A2, 2C8, 2C9, 2C19, 2E1, 2J2, 3A4	[179]	unknown	
17,18-EpETE	131339-23-6	1A1, 1A2, 2C8, 2C9, 2C19, 2D6, 2E1, 2J2, 3A4	[179]	Activated the prostacyclin receptor and sensitize TRPV1 and TRPA1 in sensory neurons Acute injection can reduce the ability to induce atrial fibrillation in mice Increase brown adipose tissue thermogenesis	[173,181,182]
5,6-DiHETE	845673-97-4	sEH	[183]	Inhibits endothelial calcium elevation during inflammation to inhibit vascular hyperpermeability TRPV4 antagonist to promote healing of colitis	[184,185]
8,9-DiHETE	867350-87-6	sEH	[183]	Exacerbated palmitic acid-induced cell death in HepG2 cells	[186]
11,12-DiHETE	867350-92-3	sEH	[183]	Associated with the liver fibrosis stage of nonalcoholic steatohepatitis	[187]
14,15-DiHETE	N/A	sEH	[183]	Negatively correlated with fatty liver index, adiposity, and metabolic syndromes in young adults	[174]
17,18-DiHETE	N/A	sEH	[183]	Negatively correlated with fatty liver index, adiposity, and metabolic syndromes in young adults	[174]
18-HEPE	141110-17-0	CYP2C, 1A2, 2B	[169]	Reduced the number of lung cancer colonies in mice when used as a pretreatment for injected B16-F0 cells, through suppression of CXCR4	[175]

## Data Availability

No new data was generated for this review.
